# Does multiparametric imaging with ^18^F-FDG-PET/MRI capture spatial variation in immunohistochemical cancer biomarkers in head and neck squamous cell carcinoma?

**DOI:** 10.1038/s41416-020-0876-9

**Published:** 2020-05-08

**Authors:** Jacob H. Rasmussen, Anders Olin, Giedrius Lelkaitis, Adam E. Hansen, Flemming L. Andersen, Helle H. Johannesen, Andreas Kjær, Ivan R. Vogelius, Lena Specht, Søren M. Bentzen, Irene Wessel, Christian von Buchwald, Barbara M. Fischer

**Affiliations:** 1 Department of Otorhinolaryngology, Head and Neck Surgery and Audiology, Rigshospitalet, University of Copenhagen, Copenhagen, Denmark; 2 Department of Clinical Physiology, Nuclear Medicine & PET and Cluster for Molecular Imaging, Rigshospitalet, University of Copenhagen, Copenhagen, Denmark; 3 Department of Pathology, Rigshospitalet, University of Copenhagen, Copenhagen, Denmark; 4 Department of Oncology, Section of Radiotherapy, Rigshospitalet, University of Copenhagen, Copenhagen, Denmark; 5 Division of Biostatistics and Bioinformatics, University of Maryland Greenebaum Cancer Center, and Department of Epidemiology and Public Health, University of Maryland School of Medicine, Baltimore, MD USA; 6 The PET Centre, School of Biomedical Engineering and Imaging Sciences KCL St Thomas’ Hospital, Westminster Bridge Road London, London, SE1 7EH UK

**Keywords:** Prognostic markers, Molecular medicine, Head and neck cancer, Cancer imaging

## Abstract

**Background:**

The purpose of this study is to test if functional multiparametric imaging with ^18^F-FDG-PET/MRI correlates spatially with immunohistochemical biomarker status within a lesion of head and neck squamous cell carcinoma (HNSCC), and also whether a biopsy with the highest FDG uptake was more likely to have the highest PD-L1 expression or the highest percentage of vital tumour cells (VTC) compared with a random biopsy.

**Methods:**

Thirty-one patients with HNSCC were scanned on an integrated PET/MRI scanner with FDG prior to surgery in this prospective study. Imaging was quantified with SUV, ADC and K^trans^. A 3D-morphometric MRI scan of the specimen was used to co-register the patient and the specimen scans. All specimens were sectioned in consecutive slices, and slices from six different locations were selected randomly from each tumour. Core biopsies were performed to construct TMA blocks for IHC staining with the ten predefined biomarkers. The spatial correlation was assessed with a partial correlation analysis.

**Results:**

Twenty-eight patients with a total of 33 lesions were eligible for further analysis. There were significant correlations between the three imaging biomarkers and some of the IHC biomarkers. Moreover, a biopsy taken from the most FDG-avid part of the tumour did not have a statistically significantly higher probability of higher PD-L1 expression or VTC, compared with a random biopsy.

**Conclusion:**

We found statistically significant correlations between functional imaging parameters and key molecular cancer markers.

## Background

Head and neck squamous cell carcinoma (HNSCC) is the most frequent malignancy in the head and neck area, and constitutes a heterogeneous disease both in regards to prognosis and tumour biology.^1–4^ The complexity of tumour biology is reflected in the hallmarks of cancer,^5^ and can be probed with histopathology using immunohistochemical (IHC) biomarkers. Numerous IHC biomarkers associated with hallmarks of cancer and prognosis have been identified. The epidermal growth factor receptor (EGFR) and Ki-67 are biomarkers associated with proliferation, the glucose transporter-1 protein (GLUT1) is associated with glucose metabolism,^6^ the biomarker p53 and Bcl-2 are associated with apoptosis, whereas the vascular endothelial growth factor (VEGF) is associated with angiogenesis and the biomarker carbonic anhydrase IX (CAIX) is associated with hypoxia.^7^ Evasion of immune destructing has been recognised as a new hallmark of cancer, and the IHC biomarker programmed death ligand 1 (PD-L1) is involved in immune escape pathways.^8,9^ All cancer-related IHC biomarkers rely on invasive tumour biopsies and in clinically practice usually on single-tumour biopsies. However, single-tumour biopsies cannot detect inter or intratumoural heterogeneity, and it is not realistic to biopsy the entire tumours to access heterogeneity nor to biopsy all lesions within a patient to assess intertumoural heterogeneity. Non-invasive modalities such as multiparametric functional imaging with positron emission tomography/magnetic resonance imaging (PET/MRI) can simultaneously provide information on FDG uptake, diffusion-weighted imaging (DWI) and dynamic contrast enhancement (DCE).^10–12^ FDG is a glucose analogue, and FDG uptake is influenced by different mechanisms, but in general FDG uptake is thought of as a surrogate for glucose metabolism.^13^ DWI images diffusion of water and is generally thought of as a surrogate for cellularity,^14^ and DCE assesses microvascular parameters and is thought of as a surrogate for perfusion or hypoxia.^15,16^ As such, PET/MRI may enable imaging of several important hallmarks of cancer^5,17,18^ simultaneously and previous studies have investigated the association between cancer-related IHC biomarkers from single-tumour biopsies and imaging features.^19,20^ However, the association between IHC biomarkers and imaging has been limited to comparisons between patients, and association between 3D functional imaging and corresponding IHC samples within patients are lacking. Ideally, the considerable intratumoural and intertumoural variability detected on functional imaging^21–23^ may complement the information from single-tumour biopsies for understanding variations across the tumour and potentially provide guidance for a suitable biopsy site. To our knowledge, no previous studies have investigated if functional imaging depicts or correlates with the spatial variability of cancer-related IHC biomarker expression in HNSCC. If a spatial correlation between functional imaging and molecular biology can be established, it will have an impact on both diagnostic methods and treatment possibilities. The purpose of this study is to investigate if preoperative functional imaging spatially correlates with IHC variability in surgical specimens.

## Methods

**Fig. 1:**
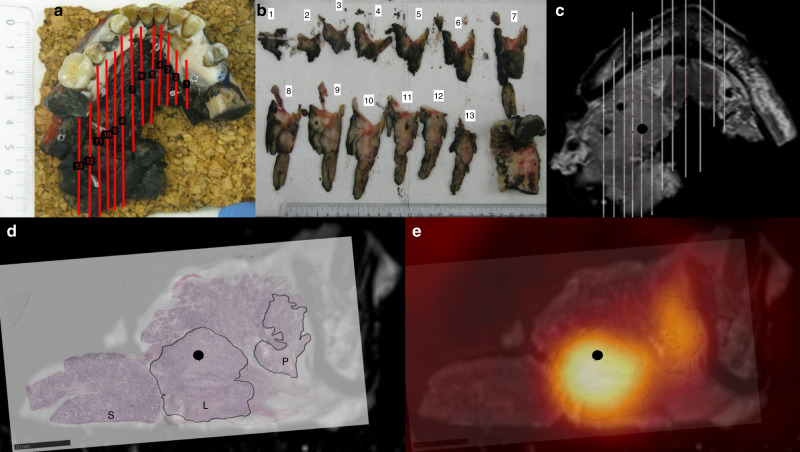
Illustration of the steps in the study from a patient with a tumour in the oral cavity and a lymph-node metastasis in level I. **a** Photograph of the specimen, the red line indicates that how the specimen was sectioned. **b** Photograph of the corresponding tumour blocks. From each block, a 4-µm section was obtained and stained with haematoxylin and eosin (H&E) and afterwards digitalised. **c** All the digitised H&E sections fused into the specimen scan, axial view. **d** A sagittal view of the pathology fused with the specimen scan. **e** The specimen scan with the pathology co-registered to the patient FDG-PET/MRI scan. The black circle in panels **c**–**e** corresponds to the same area in all three images and is where the core biopsy was taken. S: glandula submandibularis; L: lymph-node metastasis; P: primary tumour. The black contours in **d** and **e** illustrates the tumour delineated by the pathologist.

Thirty-one patients with HNSCC referred for surgery were included in this prospective study. All patients had histologically proven squamous cell carcinoma, and were referred to curative treatment with surgery for recurrent or primary HNSCC. Patients with MRI contraindications (pacemaker, metal implants not compatible with MRI) and clinically assessed tumours <1.5 cm or lymph-node metastasis <1 cm were excluded upfront. The study was approved by the local ethics committee approval number H-16049387, and the protocol published on clinicaltrials.gov (ID number NCT03160495). The workflow is explained in detail in the following list and Fig. 1. In short, we performed

- Preoperative PET/MRI scan of the patient
- Surgery and annotation of anatomical landmarks
- Formalin fixation and 3D MRI scan of specimen
- Co-registration of specimen and patient scan based on anatomical landmarks
- Specimen sectioned contiguously in tumour blocks.
- Six random tumour blocks selected for core biopsies and IHC staining
- Correlation between IHC biomarker expression and FDG uptake, diffusion and perfusion.

### PET/MRI acquisition

**Fig. 2:**
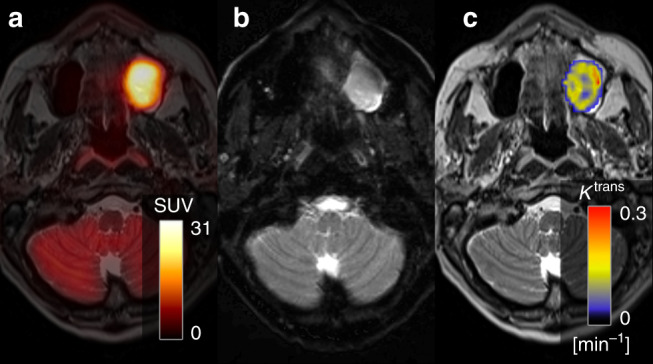
Example of multiparametric imaging with FDG uptake, diffusion and perfusion of a patient with a T2N0M0 tumour in the right maxilla involving both the maxillary sinus and the oral cavity. **a** depicts the fused FDG-PET/MRI scan, **b** depicts the diffusion-weighted imaging and **c** the dynamic contrast enhancement.

Patients were scanned on an integrated PET/MRI system (Siemens Biograph mMR) with a 3-T magnet using a head and neck coil. A detailed description of the imaging protocols can be seen in the Supplementary Material. Briefly, patients were instructed to fast for 6 h prior to the scan, and were not allowed to talk from 15 min before FDG injection until the scan was performed. Patients were scanned 60 min after FDG injection (4 MBq/kg). The reconstructed PET voxel size was 2.1 × 2.1 × 2.0 mm^3^. T1-weighted MRI after gadolinium contrast administration (Gadovist, 0.1 mM/kg body weight) and T2-weighted MRI were performed for anatomic localisation of the lesion, and morphometric imaging was performed with a 3D T2-weighted SPACE sequence with voxel size = 1.0 × 1.0 × 1.0 mm^3^. Two diffusion-weighted imaging (DWI) acquisitions were performed, one ‘standard’ DWI with a voxel size = 2.7 × 2.7 × 4.0 mm^3^, which has previously been described,^23^ and an additional DWI with a RESOLVE^24^ sequence and voxel size = 1.1 × 1.1 × 4.0 mm^3^. Dynamic contrast-enhanced (DCE) perfusion imaging utilised a 3D T1-weighted VIBE sequence with voxel size = 1.9 × 1.4 × 3.6 mm^3^. DCE images were analysed using Tissue 4D package (Siemens), which uses the model by Tofts et al.^25^ Figure 2 illustrates an example of the three functional imaging modalities from one patient in the study. Tumour was assessed on anatomical MRI and PET images by a radiologist and specialist in nuclear medicine without access to the pathology. FDG uptake was quantified using standardised uptake value (SUV),^26^ DWI was quantified using apparent diffusion coefficient (ADC),^14^ and DCE MRI was quantified using contrast transfer coefficient (K^trans^).^15,27^

### Surgery

All patients had their tumours removed *en bloc*, and anatomical landmarks were marked (by J.H.R.) per operatively in collaboration with the surgeon. The anatomical landmarks consisted of the bone, teeth, large vessels or other soft tissue structures easily identified on the preoperative patient scan. After tumour resection, the specimens were immediately fixed to cork plates with intravenous plastic cannula, and if the anatomical landmarks included soft tissue they were marked with intravenous cannula as well to identify them on the subsequent specimen MRI scan. The orientation of the tumour and the marking of anatomical landmarks were performed in collaboration and in consensus with the surgeon in the operating theatre.

### Postoperative 3D MRI scanning of the specimen

The specimen was fixed in 10% formaldehyde for 24 h, and scanned on the same PET/MRI system as the patient scan. Morphometric imaging of the specimen was acquired with a 3D T2-weighted SPACE sequence similar to the in vivo sequence, but with voxel size = 0.5 × 0.5 × 0.5 mm^3^ in a knee coil. The specimen was scanned to provide high-resolution MRI that could serve as an intermediate registration step between the patient scan (prior to surgery) and the digitalised pathology. Hence, patient MRI was registered to specimen MRI, and specimen MRI was registered to digitalised pathology. The specimens were fixated to cork plates during the formaldehyde fixation to minimise deformation in this process. The SPACE resolution employed on the specimen was enabled by the increase in signal-to-noise ratio of the knee coil.

### Co-registration of specimen and patient scan

The patient PET/MRI images were co-registered to the pathologic specimens by rigidly co-registering the 3D anatomical MRI scan of the specimen with high-resolution isotropic voxels after fixation to the patient scan in a two-step process. The first step was performed in the program register in the MINC Tool kit (McConnell Brain Imaging Center, Montreal, Canada), and included a point-by-point based alignment of the anatomical landmarks (bone, teeth or soft tissue marked with the intravenous cannula) between the patient SPACE scan and the specimen scan. The second step was performed in the software package Mirada XD General Oncology review (MIRADA Medical) that provides means of defining a transformation that relies on the visual examination of the registration. Examples are given in the Supplement Material (Supplementary Figs. S1 and S2). After visual comparison of rigid versus deformable registration—and keeping in mind the challenges of validating deformations—we chose manual rigid registration as the basis for the analysis. The quality of the registration was assessed qualitatively for all specimens, and we deemed rigid registration to be more representative and robust. After the co-registration of the patient and specimen scans, the digitalised histological images were fused to the patient PET/MRI scan as described below and illustrated in Fig. 1.

### Pathology

Before the formaldehyde fixation the specimen resection margins were marked with ink to ensure the orientation after the fixation procedure. The specimens were marked with ink in all three planes to indicate orientation and assess tumour margins. Specimens were photographed before and after inking for documentation. The specimen underwent routine formaldehyde fixation for 24 h. After the fixation and scan, the specimen was sliced in ~2–3-mm consecutive slices (Fig. 1a). The spatial location and orientation of the slices were documented with a digital imaging system (MacroPATH D_special Kit_) (Fig. 1b), and each tissue block was embedded in paraffin. From each tissue block, a 4-µm section was obtained and stained with haematoxylin and eosin (H&E). The slides were afterwards scanned at 40 × (0.23 µm/pixel) resolution with NanoZoomer XR (Hamamatsu) slide scanner or at 20 × (0.55 µm/pixel) resolution with Objective Imaging desktop scanner (Objective Imaging Ltd) and stored in Hamamatsu NDP.view format (v2.6.13), depending on the size of the specimen. The tumour area was assessed and marked on the H&E digital slides by the pathologist (Fig. 1d). The digitalised pathology was fused to the specimen scan as depicted in Fig. 1a–c. The final placement of the digitalised pathology was guided by the documented location and matching the morphology of the specimen MR image with morphology of the specimen in the digitalised pathology slice. The tumour-containing regions of interest from the digital slides were fused with the functional imaging using the co-registration of the specimen and the patient scan.

Six tumour blocks from each specimen were selected for further analysis. One block was selected from the area with the highest FDG uptake, and five blocks were selected at random within the tumour area as described in the Supplementary Material. A 3-mm wide core biopsy was performed and used to construct tissue microarray (TMA) blocks. In all, 4-µm sections from each TMA block were stained with H&E. The core biopsies were repeated up to three times if they failed due to technical reasons in the preparation of the TMA blocks. Importantly, the position of the core biopsy is known, but randomly chosen to ensure an appropriate variation across the tumour. Using the co-registration, the IHC biomarker expression can be spatially correlated to the imaging biomarkers.

Immunohistochemistry was performed on the Ventana Benchmark Ultra (Ventana Medical Systems, Tucson, AZ), Dako Omnis (Dako, Agilent Technologies, Glostrup, Denmark) and Autostainer Link 48 (Dako, Agilent Technologies, Glostrup, Denmark) platforms. The following IHC biomarkers were used: p40, p53, EGFR, Ki-67, Glut1, VEGF, Bcl-2, CAIX and PD-L1. The specific selection of IHC biomarkers was predefined and based on their association with hallmarks of cancer (EGFR, Ki-67, VEGF, Bcl-2, p53 and PD-L1), radiotherapy resistance (CAIX and EGFR) or FDG accumulation (GLUT1). P40 is a marker for squamous cell carcinoma and used with H&E in assessment of tumour cells and to estimate the tumour cell count (TCC) in each core biopsy using the Open Source Digital Pathology software program QuPath.^28^ The specific antibodies are described in the Supplementary Material. Appropriate positive controls were used, and negative control sections were incubated identically, except for the primary antibody, which was replaced by normal rabbit serum/mouse IgG. All TMA blocks were scanned at 40 × (0.23 µm/pixel) resolution with NanoZoomer XR (Hamamatsu) slide scanner.

Percentage of tumour in the core biopsy was assessed to evaluate the quality of the core, and percentage of vital tumour cells (VTC) compared with all cells in the core was reported as well as TCC. All IHC stains were scored manually as tumour proportion score (TPS) i.e., percentage of positive tumour cells in the core biopsy. Any expected (membrane, cytoplasmatic or nuclear) cell staining was visually detected, and intensity stronger than background was registered as positive. The pathologist was blinded to the information on the core biopsies (location in specimen, relation to anatomical hallmarks, data of multiparametric imaging) during the assessment process.

### Imaging region of interest definition

A region of interest (ROI) corresponding to the site and size of the core biopsy (3-mm diameter) was used to extract quantitative parameters from the three imaging modalities. The 3-mm ROI corresponds to approximately one imaging voxel, and to address a potential concern that this voxel size can be sensitive to noise, analyses were also performed by dilating the original 3-mm ROI in all dimensions to get twice the volume.

### Statistics

Partial correlation was used to analyse the association between pathology assessed with IHC biomarkers and multiparametric imaging assessed with FDG-PET, DWI and DCE using the package ppcor of R (version 3.6.0),^29^ the partial correlations are univariate with a random offset for each lesion. Confidence intervals were constructed using Bootstrapping. A Bonferroni correction was used to allow for multiple comparisons. All statistical analyses were performed in the statistical software R (version 3.6.0) and a *p*-value<0.05 was considered statistically significant. It is of clinical relevance to know if an image-guided biopsy is more representative than a random biopsy. It is nontrivial, however, to define ‘more representative' for most of the markers studied. But two scenarios are particularly interesting: (1) PD-L1 expression is used to guide the prescription of immune checkpoint inhibitors, although the cut-off values are a matter of discussion.^30^ We investigated if the highest PD-L1 expression would more likely be observed with an FDG-guided tumour biopsy than a random biopsy in the tumour; (2) similarly, for VTC we tested if the core biopsy with highest FDG uptake was more likely to harbour the highest VTC proportion than a random core biopsy. Binomial statistics was used for these analyses. Due to ties among the PD-L1 or VTC scores, the random biopsy had a higher than 1/6 chance to hit the highest PD-L1/VTC uptake, so the null hypothesis was adjusted to the random probability accounting for ties.

## Results

### Patients and lesions

In total, 31 patients were included in the study, but the data from three patients had to be excluded. The first due to technical reasons, and the second patient withdrew the consent. The third patient was excluded from the analysis because the specimen fragmented during the histological processing making it impossible to fuse the digitised pathology with imaging. Twenty-eight patients with a total of 33 lesions were available for further analysis. Two patients had five tumour blocks, and one patient only had four tumour blocks yielding 194 tumour blocks available for core biopsies. Two core biopsies were not accessible, despite repeated attempts leaving 192 core biopsies eligible for full analysis. One scan was interrupted on patient request, and only anatomical and PET imaging was available for this patient (two lesions), and in one scan, the entire lymph-node metastasis was not included in the field of view in the DWI scan, leaving 175 core biopsies available for the correlation analysis between the IHC biomarkers and the DWI scans. In four lesions, the DCE scan failed and could not be recovered, leaving 159 core biopsies available for correlation analysis between IHC biomarkers and the DCE scans. Patient and tumour characteristics can be seen in Table 1. Of the four patients with oropharyngeal tumours, one was p16 positive, and additionally, one of the three patients with only an excised lymph-node metastasis was p16 positive.

**Table 1:** Patient characteristics.

Characteristics	Value (%)
*Age*	
Median	63
Range	50–80
*Sex*	
Male	16 (57)
Female	12 (43)
*Tumour subsite*	
Oral cavity	16
Oropharynx	4
Hypopharynx/larynx	3
Maxillary sinus	2
Lymph node	3 (+5)^a^
*Recurrence*	
Yes (former radiotherapy)	10
No	18
*Smoking*	
Never	3
Former	15
Current	10
*Time interval between PET/MRI and surgery in days*	
Median	3
Range	1–11

^a^Five patients had a lymph node beside the primary tumour yielding 33 lesions.

### Pathology

Figure 1 illustrates the different steps in workflow: the section of the specimen and the corresponding tumour blocks (Fig. 1a, b), the image fusion with the microscopic pathology (Fig. 1c, d), and an example of FDG-PET/MRI from the patient scan co-registered with pathology (Fig. 1e). Fifteen lesions were scanned both before and after formalin fixation to assess tumour shrinkage. The specimens shrank 4.8 cm^3^ in average (range 1.2–8.2).

### Imaging

Figure 2 illustrates the multiparametric imaging with the three different imaging modalities from a patient, FDG uptake (Fig. 2a), diffusion (Fig. 2b) and perfusion (Fig. 2c). The DWI with the RESOLVE sequence did in some cases show image distortion and signal drop out. As a consequence, only the results from the ‘standard’ DWI acquisition were used in the spatial correlation analysis. Unsurprisingly, considering the small size of the TMA cores, there was essentially no variation in imaging metrics across the cores (Supplementary Material Fig. S3). Hence, SUV_mean_ and ADC_mean_ were used in the correlation analyses. For DCE perfusion, the most frequent measure reported in the literature is the transfer coefficient K^trans15^, and this was used in the subsequent correlation analyses.

### Correlation analysis

**Fig. 3:**
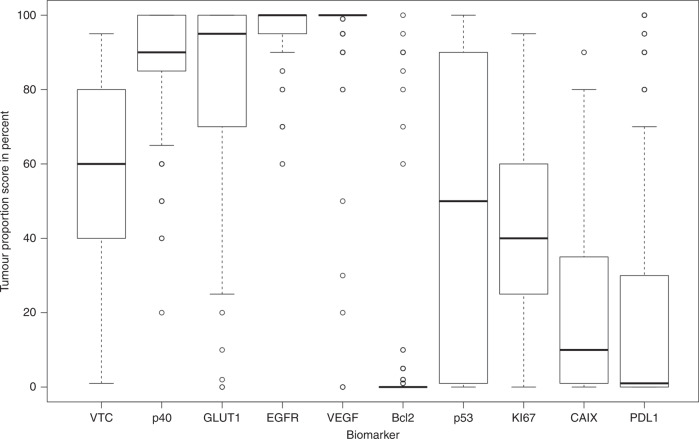
Boxplot illustrating the heterogeneity of vital tumour cells (VTC) and the IHC biomarkers in each of the 194 cores from all 33 lesions. All biomarkers were scored in percent as tumour proportion score in the core biopsies.

Figure 3 depicts a boxplot illustrating the variation of the IHC markers in percentage of VTC in the core and the TPS of the different IHC biomarkers in all 194 cores. The IHC biomarkers p53, Ki-67, CAIX and PD-L1 showed most variation between all core biopsies, whereas VEGF and Bcl-2 showed little variation (Fig. 3).

**Fig. 4:**
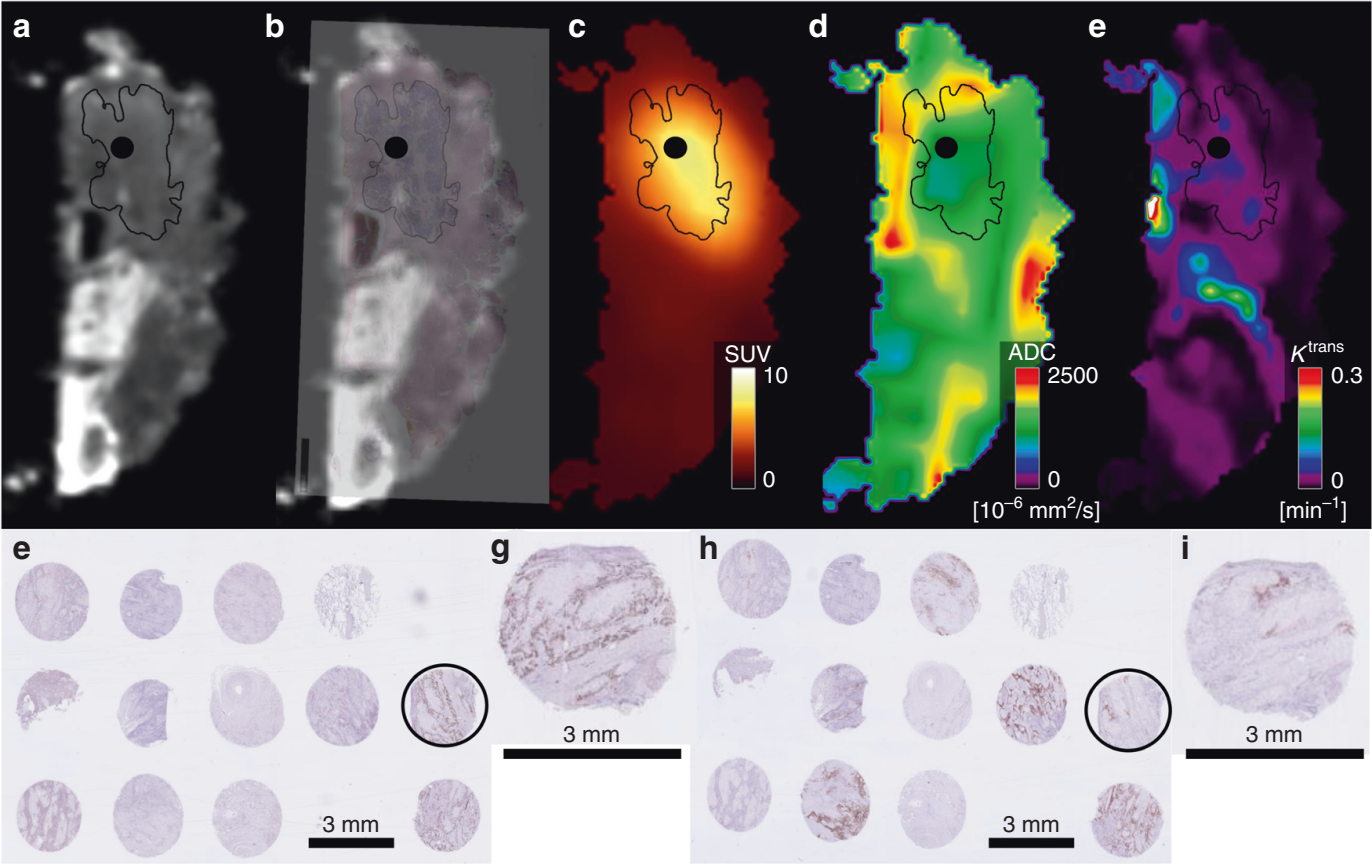
Example of the functional imaging and pathology from a patient with a lymph node metastasis. **a** An image from the 3D-morphometric scan of the specimen. **b** The 3D-morphometric scan fused with the digitalised pathology. **c** The FDG-PET scan. **d** The DWI scan. **e** The DCE scan. The black contour illustrates the tumour defined by the pathologist, and the black circle represents the areas where the core biopsy was taken. **f** illustrates an example from the TMA block stained with p53. The black circle depicts the core biopsy shown above (**a**–**e**). In panel **g**, the core biopsy is magnified. **h** shows the same TMA stained for CAIX, and in panel **i** the core biopsy is magnified.

Figure 4 shows the functional imaging from a patient, where the core biopsy was taken (Fig. 4a–e), and the corresponding pathology for two biomarkers, p53 and CAIX (Fig. 4f–i).

Table 2 shows the results from the partial correlation analysis with 95% confidence intervals. FDG uptake correlated positively with both percentage of VTC and tumour cell count in the biopsy cores, and there was a significantly positive correlation between the hypoxic marker CAIX and FDG uptake. There was a significant and positive correlation between DWI and the apoptotic IHC marker p53 and DWI correlated significantly and negatively with the proliferation IHC marker Ki-67, the apoptotic IHC biomarker Bcl-2 and with the immune checkpoint inhibitor PD-L1. Moreover, K^trans^ correlated positively with p53. However, despite being significant, all correlations were weak at best. After correction for multiple comparisons with the Bonferroni correction, the correlation between FDG uptake and VTC was significant as were the correlations between diffusion (ADC) and Ki-67, PD-L1 and Bcl-2.

**Table 2:** Partial correlation coefficients.

	SUV_mean_		ADC_mean_		K^trans^	
	Correlation coefficient (95% CI)	*p*-value	Correlation coefficient (95% CI)	*p*-value	Correlation coefficient (95% CI)	*p*-value
P40	−0.032 (−0.172–0.104)	0.656	0.054 (−0.099–0.192)	0.472	−0.038 (−0.184–0.128)	0.631
VTC	0.273 (0.119–0.412)	**<0.001**^a^	−0.055 (−0.204–0.102)	0.469	−0.070 (−0.232–0.084)	0.379
TCC	0.184 (0.041–0.314)	**0.010**^a^	−0.043 (−0.189–0.128)	0.565	−0.011 (−0.172–0.144)	0.886
GLUT1	−0.044 (−0.182–0.096)	0.547	0.117 (−0.046–0.250)	0.118	−0.035 (−0.184–0.122)	0.667
EGFR	0.071 (−0.073–0.209)	0.326	−0.060 (−0.199–0.093)	0.420	−0.038 (−0.191–0.111)	0.637
P53	0.002 (−0.130–0.152)	0.980	0.190 (0.039–0.323)	**0.010**^a^	0.193 (0.037–0.339)	**0.015**^a^
Ki-67	0.058 (−0.095–0.218)	0.428	−0.253 (−0.387 to −0.106)	**<0.001**^a^	−0.020 (−0.173–0.122)	0.800
CAIX	0.195 (0.050–0.320)	**0.007**^a^	0.021 (−0.114–0.153)	0.781	0.006 (−0.141–0.163)	0.940
PD-L1	0.129 (−0.008–0.274)	0.075	−0.246 (−0.380 to −0.094)	**<0.001**^a^	0.071 (−0.089–0.236)	0.373
VEGF	−0.065 (−0.215–0.075)	0.369	−0.019 (−0.162–0.147)	0.802	−0.022 (−0.173–0.128)	0.788
Bcl-2	0.140 (0.005–0.271)	0.054	−0.283 (−0.410 to −0.125)	**<0.001**^a^	0.061 (−0.120–0.202)	0.448

*VTC* percentage vitale tumour cells, *TCC* tumour cell count.

Significant values are in bold and marked with superscript ^a^.

Correlation plots for the significant correlations, in order of PET-positive tumour volume, can be assessed in Supplementary Fig. S4. The PET-positive tumour volume was not associated with the correlation between imaging parameters and IHC markers.

The sensitivity analysis using the larger ROI with twice the volume of the 3-mm ROI showed the same results for FDG uptake (SUV) and diffusion of water (ADC), but K^trans^ did not correlate significantly with PD-L1. The results can be seen in the Supplementary Material Table S1.

We tested whether the core biopsy with highest FDG uptake was more likely to have the highest PD-L1 expression or highest VTC compared with a random biopsy. In 14 of the 33 lesions (42%; 95% CI: 25–61%), the core biopsy with highest FDG uptake had the highest PD-L1 expression compared with 39% when the biopsy was performed random (*p* = 0.72). For VTC, 10 of the 33 (30%; 95% CI: 16–49%) lesions with highest FDG uptake had highest VTC expression compared with 25% with a random biopsy (*p* = 0.55).

## Discussion

To the best of our knowledge, this is the first study to assess spatial correlation between multiparametric functional imaging and IHC of cancer biomarkers. The long-term goal would be to facilitate the estimation of pathological intratumoural variation, here through IHC markers, from a non-invasive functional imaging procedure. We also address the question if functional imaging could guide the site for biopsies. This could not be confirmed in the present analysis as a biopsy taken from the most FDG avid part of the tumour did not have a statistically higher probability of having the highest PD-L1 expression or VTC, compared with a random biopsy. However, it should be emphasised that in the analysis of our current data, all the biopsies were selected to be representative of vital tumour tissue on the pathological slides. In other words, we specifically excluded cores without useful tumour tissue, and it is therefore conceivable that image-guided biopsies obtained from a patient would be more likely to contain tumour cells than a biopsy based on conventional clinical examination. As such, image-guided biopsies might still provide clinical relevance when the biopsy is performed from a tumour within a patient. Furthermore, more specific tracers than FDG or more specific functional MRI procedures could hopefully improve on the situation observed here.

The spatial and positive correlations between FDG uptake on one hand and percentage of VTC, tumour cell count and the hypoxic marker CAIX on the other were positive and significant. This is encouraging and makes sense from a simple mechanistic biologic point view. It seems plausible that areas with more vital tumour cells consume more glucose, and it is well known that glucose transport is higher in hypoxic regions. However, with IHC staining only vital cells (alive prior to formalin fixation) can be assessed, and cellular viability is required for the expression of CAIX or any of the ICH biomarkers investigated here. On the other hand, all core biopsies were performed from areas with representative tumour tissue. The significant correlations between FDG uptake and percentage of VTC and tumour cell count emphasise that although FDG is a broad tracer for glucose metabolism it does seem to reflect tumour burden, metabolism and is associated with hypoxia (CAIX). Besides surgery, radiotherapy is an important treatment modality for HNSCC patients; however, hypoxic tumours are more resistant to radiotherapy. A correlation between a non-invasive hypoxic imaging modality and a biologic hypoxic biomarker such as CAIX could be used both as a possible prognosticator and as a predictor for early treatment response during radiotherapy.^31^ FDG uptake as a hypoxic marker has been investigated both in preclinical and in clinical studies,^32^ in different tumour types,^33^ and in HNSCC.^34–36^ Still, the results from different studies are ambiguous as illustrated in a recent meta-analysis.^20^ None of the studies above investigated spatial correlation between imaging biomarkers and biologic biomarkers as presented in this study, and a spatial correlation is a necessity if FDG or any other imaging biomarker is to be used as a possible target in dose painting radiotherapy^37^ or as a predictor for relapse location.

There is no obvious biologic explanation for the significant spatial positive correlation between the apoptotic IHC marker p53 and diffusion (ADC) and the negative spatial correlation between Bcl-2 and ADC. However, Bcl-2 was homogenously expressed in all cores (Fig. 3), and Supplementary Fig. S4 in the Supplementary Material illustrates that the negative correlation is driven by few lesions. The biomarker p53 is a tumour suppressor involved in apoptosis, and p53 expression has shown mixed prognostic effects in HNSCC with high expression of p53 associated with an increased the risk of locoregional failure, but a decreased risk of distant metastasis.^38^ The biomarker p53 was heterogeneously expressed (Fig. 3; Supplementary Fig. S4), and the positive spatial correlation between ADC and p53 could suggest that ADC is sensitive to changes in cell morphology during apoptosis^31^ and the spatial correlation between K^trans^. Furthermore, the spatial correlation between p53 and K^trans^ could indicate that areas prone to apoptosis (high p53 expression) have more capillary permeability (high K^trans^). The proliferation IHC marker Ki-67 correlated negatively with ADC and illustrates that areas with low diffusion of water have a higher proliferation index compared with areas with high diffusion, likely reflecting the increased cellularity resulting from high proliferation (Supplementary Fig. S4). This is in line with previous studies and a recent meta-analysis,^20^ reporting an inverse correlation between Ki-67 and ADC in different tumour types, including HNSCC.^39–42^ Moreover, previous studies have shown different results regarding correlation between ADC and cellularity. There was an inverse correlation in most tumour types, including HNSCC,^43^ and DWI is generally considered a surrogate for cellularity. Here, we find a weak negative correlation between ADC and cellularity expressed as VTC or TCC, but the results were not significant. Importantly, to our knowledge, previous studies have tested correlation between a single-tumour biopsy taken from an unknown origin in the tumour and an imaging parameter from either somewhere else in the tumour or from the entire tumour, such as ADCmin or ADCmean. As such these studies provide neither information on spatial correlation nor intratumoural variations.

The negative spatial correlation between the immune checkpoint inhibitor PD-L1 and ADC and positive spatial correlation between PD-L1 and K^trans^ is intriguing in the context of immunotherapy. Such associations suggest that it may be possible to define multiplexed PD-L1 point biopsy and imaging signatures, which are superior at identifying patients likely to benefit from immunotherapy as compared with a single biopsy alone.^30^ The observed difference in the sign of the correlation between IHC markers and FDG uptake (positive correlation with VTC and CAIX) on one hand, and ADC (negative correlation with p53, Ki-67 and PD-L1) on the other is interesting, but suggests that the two imaging modalities may convey complementary tissue characterisation, leading to the hypothesis that FDG describes the metabolism and tumour microenvironment (hypoxia), whereas ADC seems to correlate with more specific malignant characteristics. In this study, it is not evident that DCE provides any additional information not available from FDG-PET or DWI.

The prospective design, logistic setup with 3D-morphometric MRI of the specimen on the same scanner, the repeated results from the sensitivity analysis with different ROI sizes and the relatively large patient population with multiple biopsies are strengths in this study. Both patients with newly diagnosed disease and patients with recurrence were included, and the patients had different tumour subsites within the head and neck region. This diversity was obviously a study design choice to allow sufficient accrual, but it is also a weakness, as site-specific correlations could be speculated to exist, but vanish in the diversity of patients. However, as demonstrated by the weak correlations and observed confidence intervals evident from Table 2 and Supplementary Fig. S4, the putative existence of a strong correlation between the studied imaging and IHC biomarkers in a clinically defined subset of HNSCC patients seems unlikely. A more relevant criticism is that co-registration—despite all our efforts—will include some misalignment and thus diminish the apparent correlations in our analysis. Moreover, although this is a relatively large study sample considering the logistically challenging setup the sample size is limited. Nevertheless, from the confidence intervals in Table 2, we conclude that a strong correlation between the IHC cancer biomarkers and imaging features is unlikely.

In conclusion, the statistically significant spatial correlations between functional imaging parameters and key molecular cancer markers is encouraging. However, the modest correlation challenges the direct clinical applicability and it indicates that further efforts are needed to elucidate exactly how functional imaging parameters can be used to quantify intratumoural heterogeneity.

## Supplementary information

Supplemental material

## Data Availability

The data set used and analysed during the current study is available from the corresponding author on reasonable request.
