# Supplementary material for: Does multiparametric imaging with 18F-FDG-PET/MRI capture spatial variation in immunohistochemical cancer biomarkers in head and neck squamous cell carcinoma?
Source: Br J Cancer. 2020 May 8;123(1):46–53. doi: 10.1038/s41416-020-0876-9 (PMC7341803; doi:10.1038/s41416-020-0876-9)
Supplement: Supplementary file 1 — Supplemental material [file 41416_2020_876_MOESM1_ESM.docx]

**Supplementary material**

*PET/MRI acquisition*

Patients were scanned 60 minutes after FDG injection (4 MBq/kg) and all scans were performed with gadolinium contrast. PET was performed as a single-bed, 20-min acquisition 60 and again 90 minutes after the FDG injection. PET data were reconstructed using ordinary Poisson ordered-subset expectation maximization with 3 iterations, 24 subsets, and 4 mm gaussian post processing filter into 344 x 344 matrices. The reconstructed voxel size was 2.1×2.1×2.0 mm^3^. Resolution modeling (point spread function) was not applied.

T1 and T2 weighted imaging was performed for anatomic localization of the lesion, and morphometric imaging was performed with a 3D T2 weighted SPACE sequence with matrix size = 320 x 320 x 100, voxel size = 1.0 x 1.0 x 1.0 mm^3^, TR/TE = 1600/110 ms, parallel imaging factor = 3, flip angle = 100º. Diffusion Weighted Imaging (DWI) acquisition was based on single-shot EPI with matrix size = 92 x 92 x 40, voxel size = 2.7 x 2.7 mm^2^, slice thickness/gap = 4.0/0.4 mm, TR/TE = 3000/84 ms, parallel imaging factor = 4, flip angle = 90º and b-values (0, 800 s/mm^2^). The standard DWI was measured as 5 stacks of 8 slices each. For field mapping, double echo sequences with a repetition time of 100 ms and echo times of 4.92 and 7.38 ms. DWI distortions were corrected using the FUGUE algorithm^1^ (Analysis Group, FMRIB, Oxford, UK). An additional DWI acquisition was performed and utilized a readout-segmented, multi-shot Echo-Planar Imaging (EPI) RESOLVE^2^ sequence with matrix size = 192 x 192 x 25, voxel size = 1.1 x 1.1 mm^2^, slice thickness/gap = 4.0/1.2 mm, shots = 9, TR/TE = 5600/61 ms, parallel imaging factor = 2, flip angle = 180º and b-values (0, 800 s/mm^2^). Dynamic contrast enhanced (DCE) perfusion imaging utilized a 3D T1-weighted VIBE sequence with matrix size = 192 x 138 x 20, voxel size = 1.9 x 1.4 x 3.6 mm^3^, TR/TE = 5.18/1.78 ms, parallel imaging factor = 2, flip angle = 15º and 34 dynamic images of duration 7.8 s. MRI contrast (Gadovist, 0.1 mM/kg body weight) was administered with the 5^th^ dynamic image. For tissue T1-mapping, four VIBE sequences were acquired with different flip angles = 2º, 6º, 10º, 15º and with the abovementioned imaging parameters. DCE images were analyzed on a voxel-basis over the entire patient image volume using Tissue 4D package (Siemens). The software uses the model by Tofts^3^ to estimate, the pharmacokinetic parameters K^trans^, v_e_ and k_ep_, representing transport from plasma to interstitial space, extravascular extracellular space and transport from extravascular extracellular space back to plasma, respectively. The model is based on a population based arterial input function and a tissue T1-mapping, which is derived by the software given the MRI sequences acquired at different flip angles. The motion correction, which is also implemented on the software, was applied if motion was evident based on visual inspection of the dynamic series.

*Selection of tumor blocks and immunohistochemistry*

Six tumor blocks from each lesion were selected for further histological processing. One tumor block was selected from the most FDG avid area of the tumor and the remaining five were selected at random to avoid potential selection bias. The randomization was performed with a random number generator develop in the statistical software R (version 3.4.1).

The following antibodies were used for immunohistochemical testing:

- p40 (platform Dako Omnis, clone BC28, code ACI3066C, mouse monoclonal anti-human, 1 + 50, Biocare Medical, Pacheco CA, USA),
- p53 (platform Benchmark Ultra, clone DO-7, code 800-2912, mouse monoclonal anti-human, read-to-use, Roche, Hvidovre, Denmark),
- Epidermal growth factor receptor, (EGFR, platform Benchmark Ultra, clone 5B7, code 790-4347, rabbit monoclonal anti-human, ready-to-use, Roche),
- Ki-67 (platform Dako Omnis, clone MIB-1, code IR626, mouse monoclonal anti-human, ready-to-use, Dako),
- Glut1 (platform Benchmark Ultra, clone SPM498, code MS-10637-P, mouse monoclonal anti-human, 1 + 100, Thermo Scientific, Waltham, MA USA),
- Vascular Endothelial Growth Factor (VEGF, platform Autostainer Link 48, polyclonal, code RB 9031-P-A, rabbit polyclonal anti-human, ready-to-use, Thermo Scientific),
- Bcl-2 (platform Benchmark Ultra, clone 124, code 790-4464, mouse monoclonal anti-human, ready-to-use, Roche),
- Carbonic Anhydrase IX (CAIX, platform Benchmark Ultra, clone TH22, code NCL-L-CAIX, mouse monoclonal anti-human, 1 + 75, Novocastra, Ballerup, Denmark),
- Programmed death-ligand 1 (PD-L1, platform Autostainer Link 48, clone 28-8, pharmDx kit, rabbit monoclonal anti-human, 1 + 200, Dako).

References

1. Hansen AE, Rasmussen J, Johannesen HH, et al. Geometric distortions of diffusion weighted imaging of the head/neck in combined PET/MR: optimization of image acquisition and post-processing correction for oncology applications. *EJNMMI Phys*. 2014;1(Suppl 1):A76. doi:10.1186/2197-7364-1-S1-A76

2. Porter DA, Heidemann RM. High resolution diffusion-weighted imaging using readout-segmented echo-planar imaging, parallel imaging and a two-dimensional navigator-based reacquisition. *Magn Reson Med*. 2009;62(2):468-475. doi:10.1002/mrm.22024

3. Tofts PS, Brix G, Buckley DL, et al. Estimating kinetic parameters from dynamic contrast‐enhanced t1‐weighted MRI of a diffusable tracer: Standardized quantities and symbols. *J Magn Reson Imaging*. 1999;10(3):223-232. doi:10.1002/(SICI)1522-2586(199909)10:3<223::AID-JMRI2>3.0.CO;2-S

*Supplementary figures*

**Figure S1.** Example of the co-registration from one of the patients. To the left the specimen scan axial (top left) and coronal (bottom left) view. To the right the co-registration of the patient and specimen scans. The orange is the specimen scan. Top right axial view and bottom right coronal view.


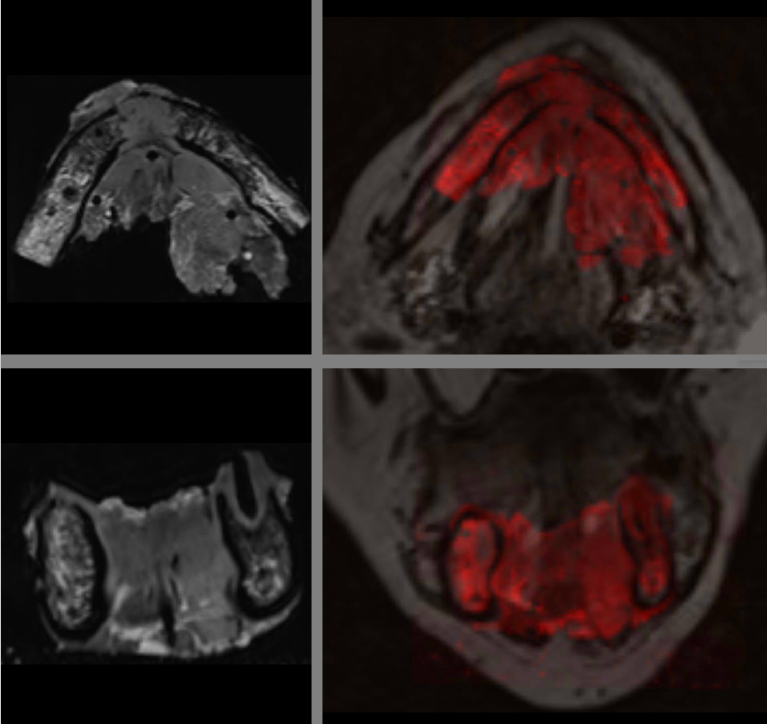


**Figure S2.** Example of the co-registration of the specimen scan and patient scan in one of patients. The top panel shows the point by point based registration with two points in the axial view. The orange is the specimen. In the top left panel is the patient MRI scan (axial view). In the top middle panel is the specimen scan (axial view), and in the right top panel is the co-registration of the specimen and the patient scan (axial view). The bottom panel shows the result of the co-registration in both axial, coronal and sagittal view, the orange is the specimen.


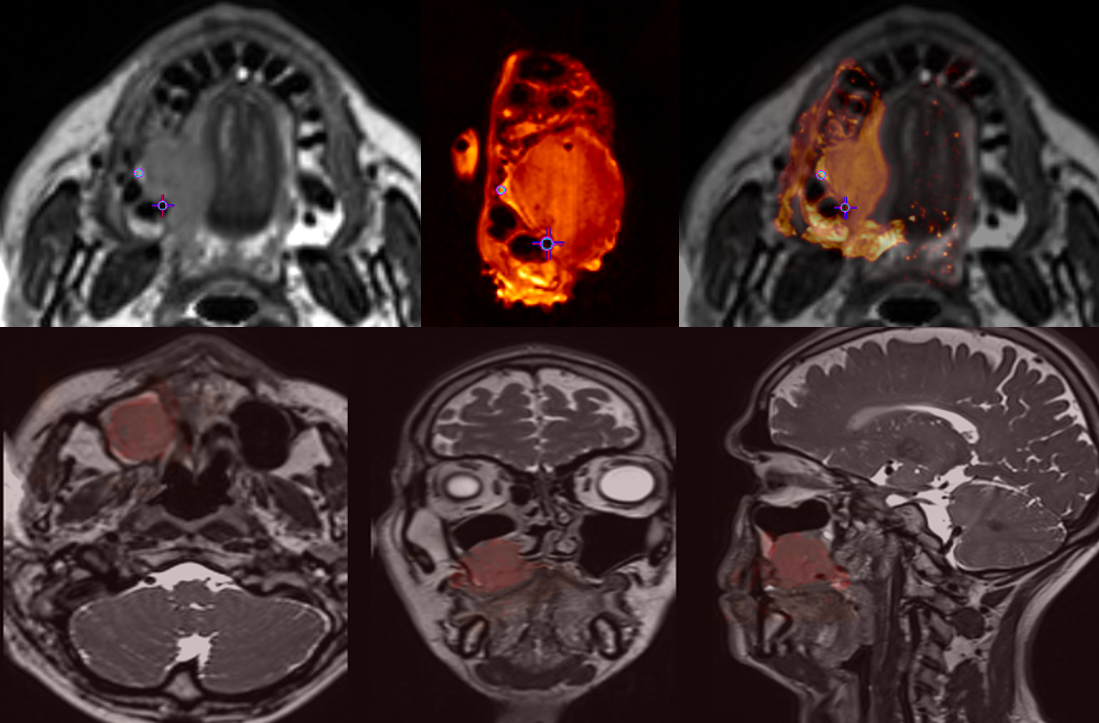


**Figure S3.** Scatter plot of SUV_max_ and SUV_mean_; ADC_min_ and ADC_mean_ and Ktrans_mean_ and Kep_mean_ in each core biopsy.


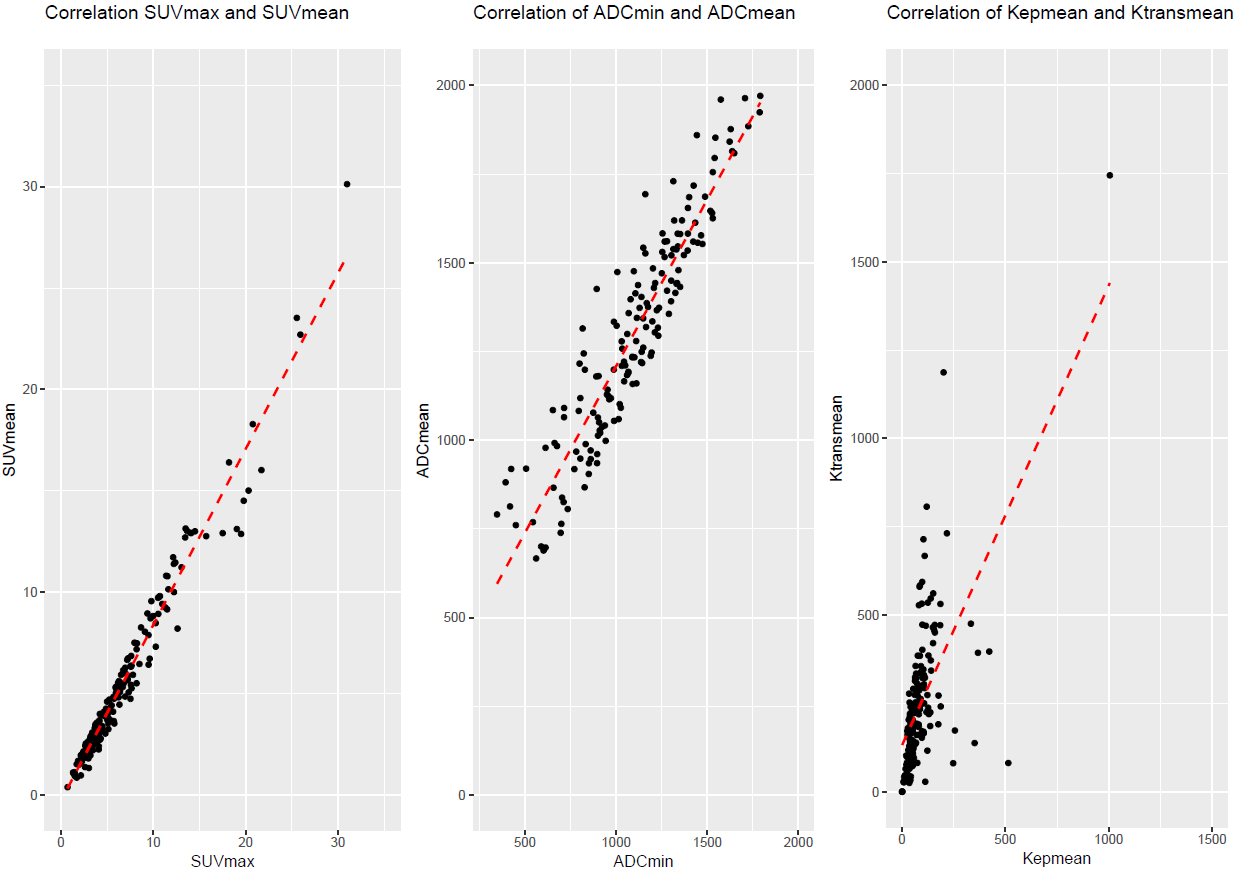


**Figure S4.** Correlation plot for all 33 lesions between percentage of vital tumor cells (VTC) and the IHC biomarkers CAIX, p53, Ki-67, PD-L1, Bcl-2 and imaging biomarkers ADC and Ktrans that were significant in the partial correlation analysis.


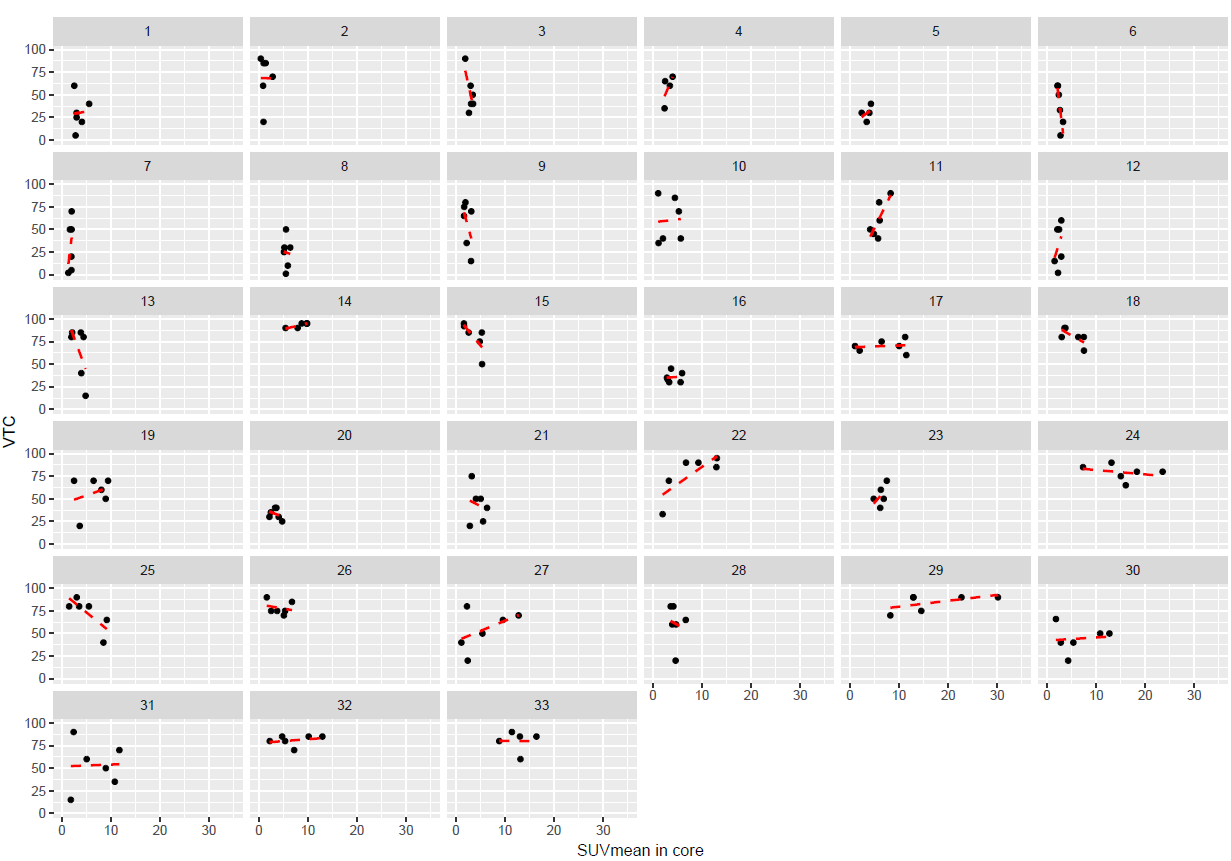


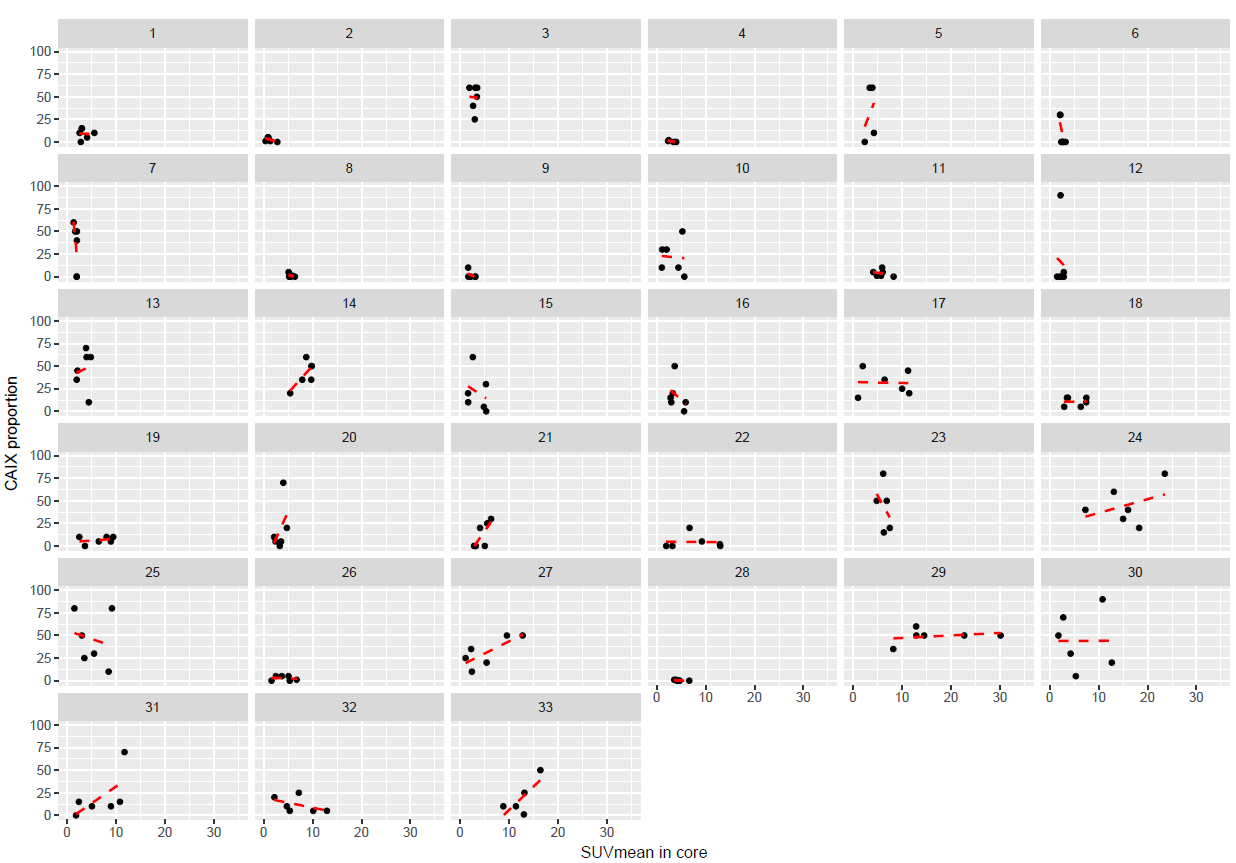


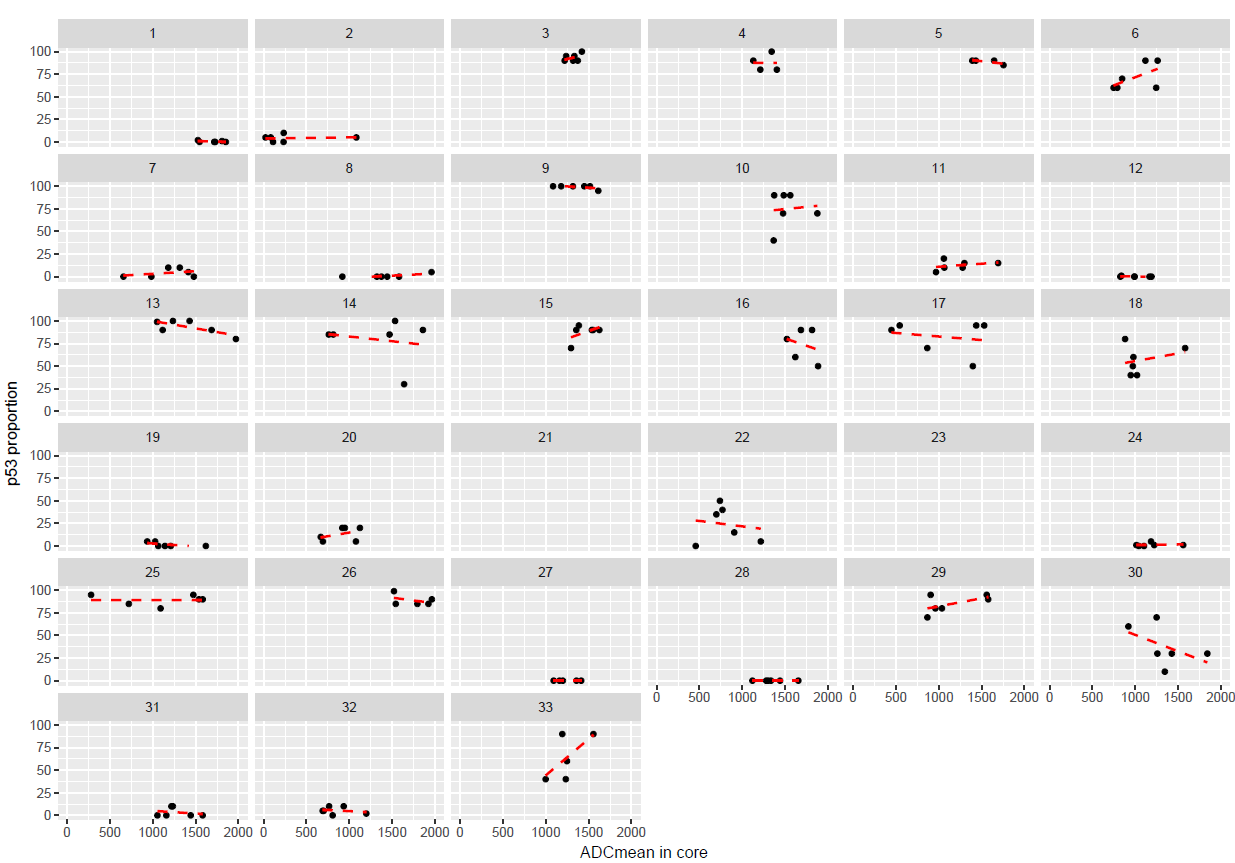


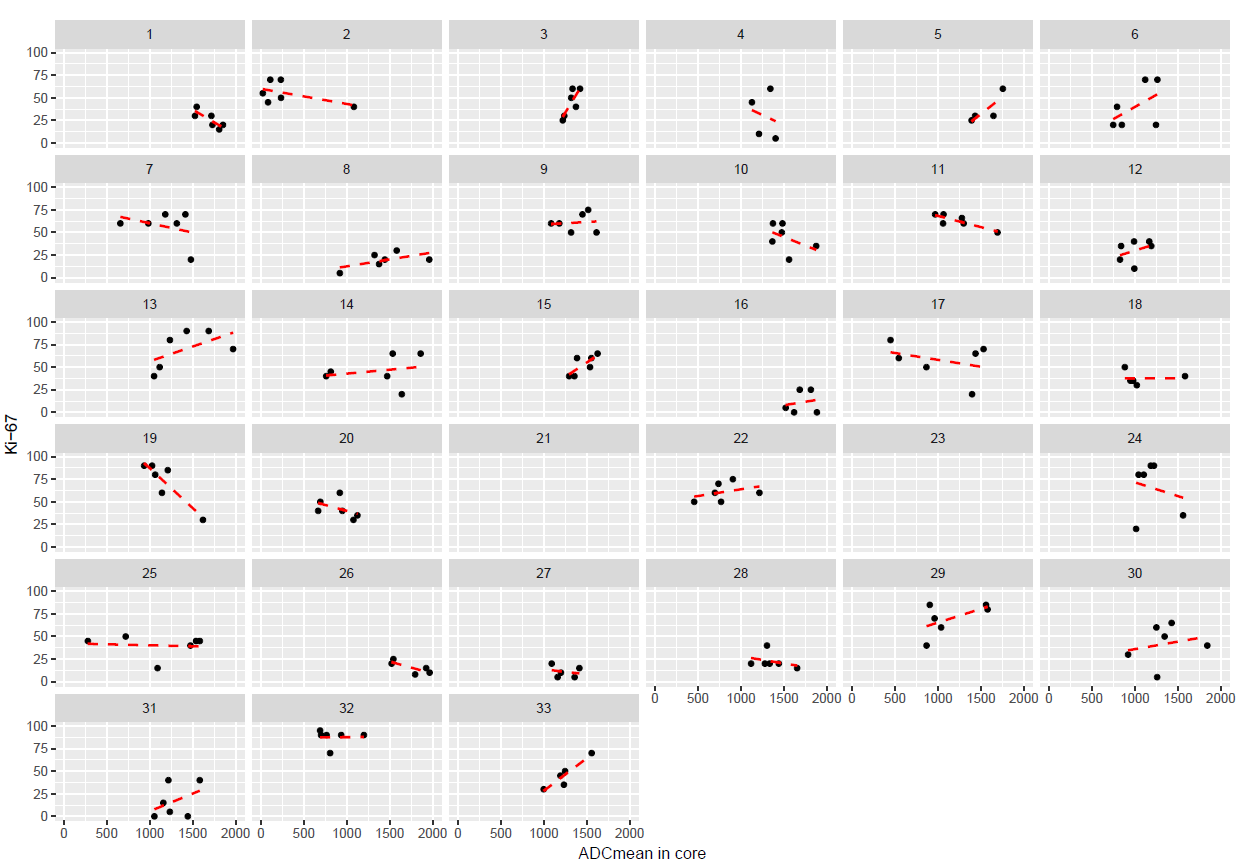


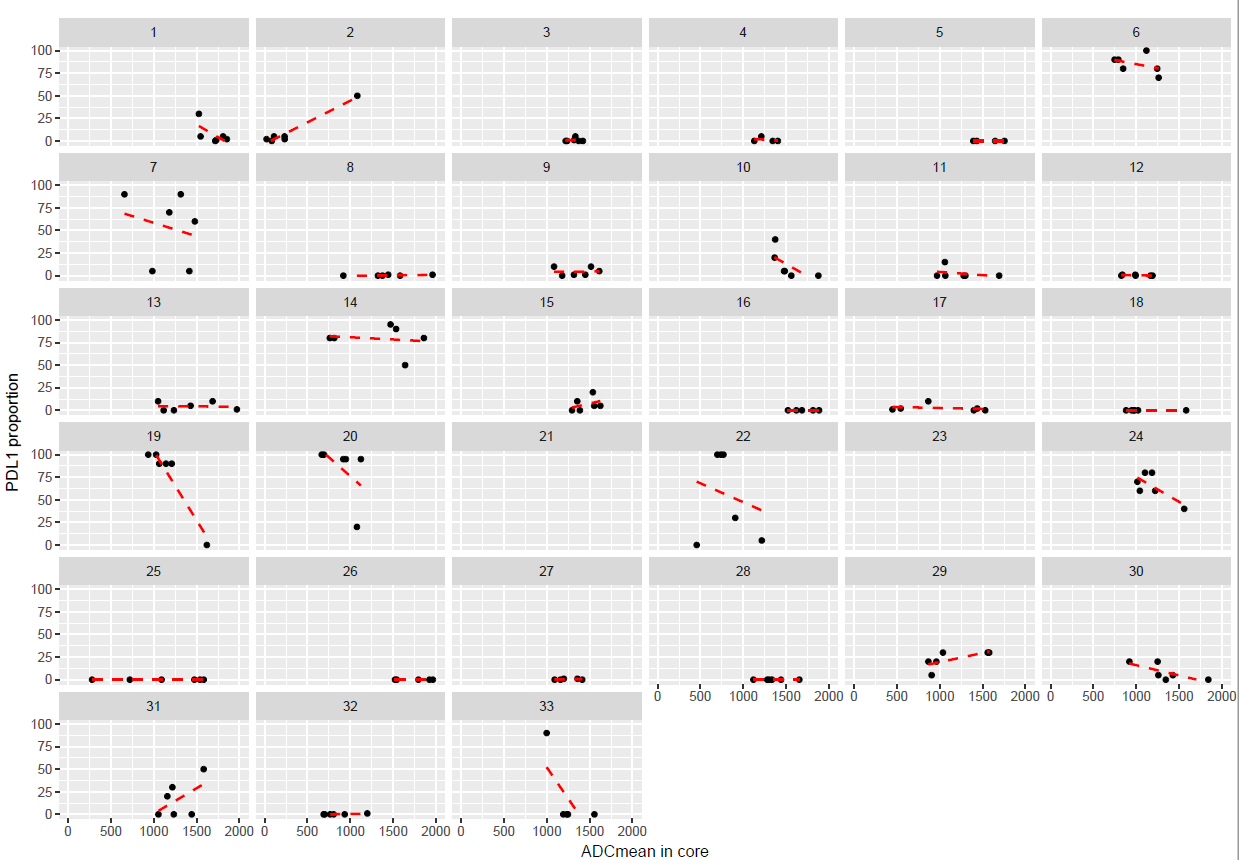


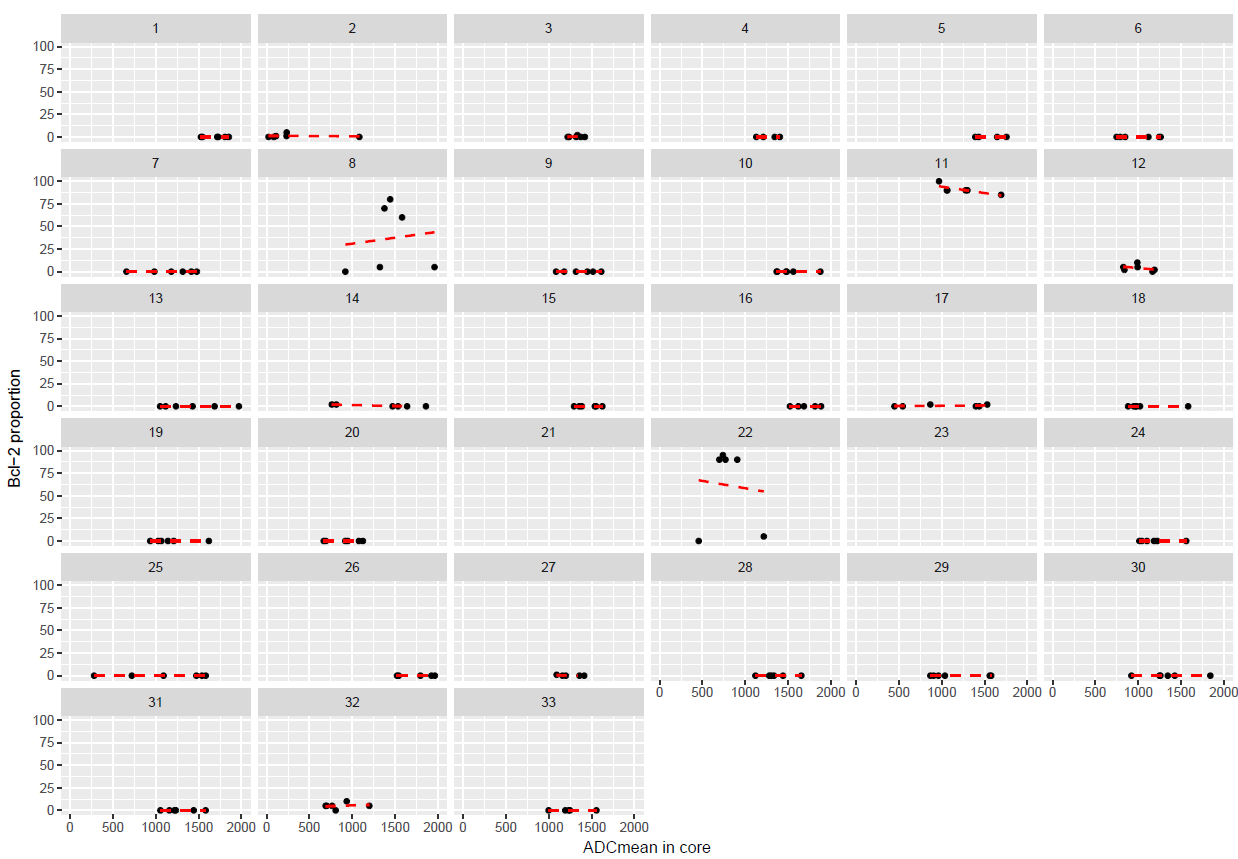


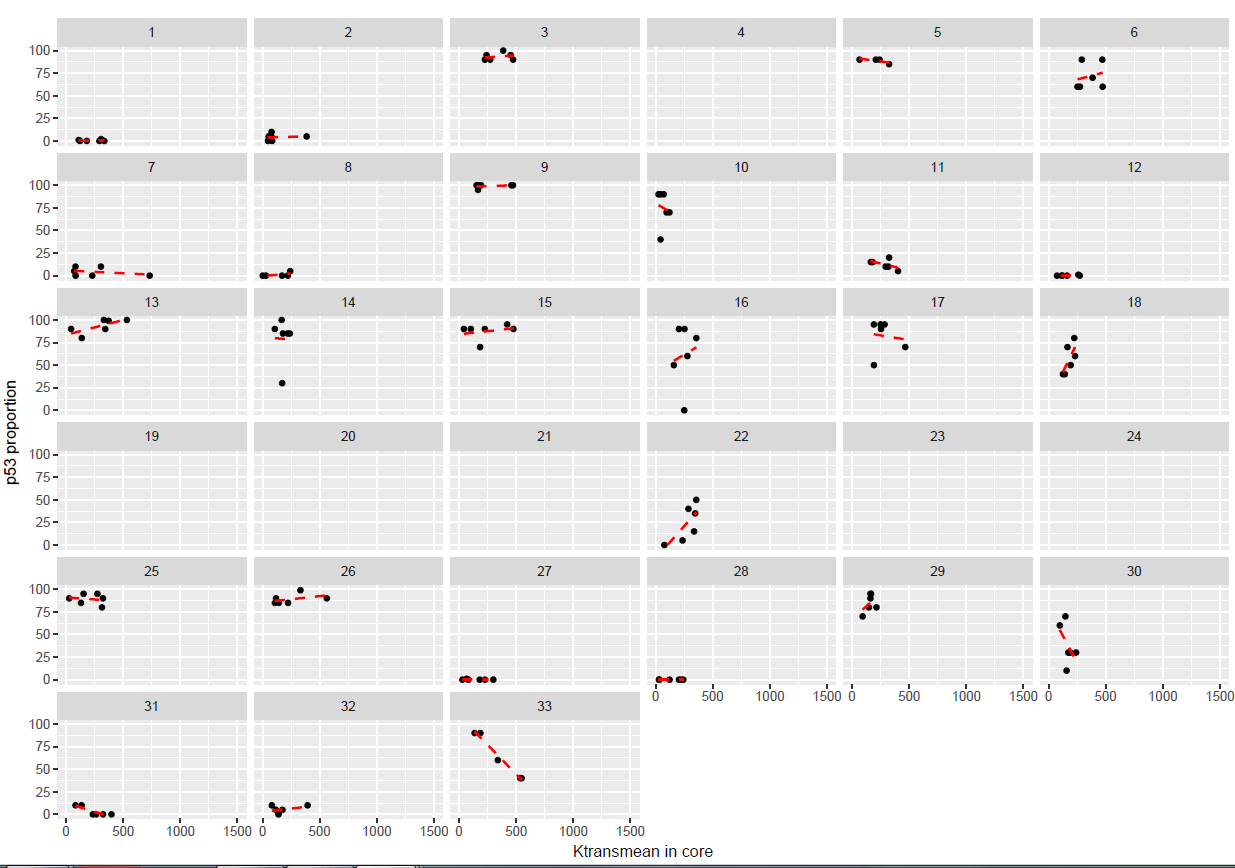


**Table S1.** Partial correlation coefficients between IHC markers and imaging parameters from a ROI of 6-mm in diameter. Significant values are in bold and marked with *.

|  | **SUV_mean_** | | **ADC_mean_** | | **K^trans^** | |
| --- | --- | --- | --- | --- | --- | --- |
|  | Correlation coefficient | p-value | Correlation coefficient | p-value | Correlation coefficient | p-value |
| **P40** | -0.031 | 0.672 | 0.060 | 0.420 | -0.062 | 0.411 |
| **VTC** | 0.275 | **<0.001*** | -0.058 | 0.437 | -0.030 | 0.687 |
| **GLUT1** | -0.045 | 0.533 | 0.112 | 0.134 | 0.027 | 0.717 |
| **EGFR** | 0.070 | 0.335 | -0.043 | 0.570 | -0.022 | 0.771 |
| **P53** | 0.001 | 0.992 | 0.187 | **0.012*** | 0.243 | **0.001*** |
| **Ki67** | 0.057 | 0.430 | -0.257 | **<0.001*** | -0.055 | 0.465 |
| **CAIX** | 0.199 | **0.006*** | 0.023 | 0.757 | 0.008 | 0.909 |
| **PD-L1** | 0.128 | 0.077 | -0.261 | **<0.001*** | -0.104 | 0.163 |
| **VEGF** | 0.067 | 0.355 | -0.019 | 0.803 | 0.100 | 0.179 |
| **Bcl-2** | 0.139 | 0.055 | -0.276 | **<0.001*** | 0.172 | **0.021*** |
